# Supplementary material for: SLC35D3 promotes white adipose tissue browning to ameliorate obesity by NOTCH signaling
Source: Nat Commun. 2023 Nov 23;14:7643. doi: 10.1038/s41467-023-43418-5 (PMC10667520; doi:10.1038/s41467-023-43418-5)
Supplement: Supplementary file 1 — Supplementary Information [file 41467_2023_43418_MOESM1_ESM.pdf]

Supplementary information

Title: SLC35D3 promotes white adipose tissue browning to ameliorate obesity by NOTCH signaling

Hongrui Wang[#1], Liang Yu[#1], Jin’e Wang[#2], Yaqing Zhang[2], Mengchen Xu[1], Cheng Lv[1], Bing Cui[1], Mengmeng Yuan[1], Yu Zhang[1], Yupeng Yan[1], Rutai Hui[1], Yibo Wang[*1]

1, State Key Laboratory of Cardiovascular Disease, Fuwai Hospital, National Center for Cardiovascular Diseases, Chinese Academy of Medical Sciences and Peking Union Medical College, Beijing, China.

2, College of Medical Science, China Three Gorges University, Yichang, Hubei, China.

#, these authors contributed equally: Hongrui Wang, Liang Yu, Jin’e Wang.

***Correspondence:** Yibo Wang, State Key Laboratory of Cardiovascular Disease, National Center for Cardiovascular Diseases, Fuwai Hospital, Chinese Academy of Medical Sciences and Peking Union Medical College, 167 Beilishi Rd, Beijing, 100037, China. E-mail: yibowang@hotmail.com.

Supplementary Figure 1. Adipose-specific *Slc35d3* knockout induced adipose tissue inflammation and liver steatosis in mice.

a qPCR analysis of *F4/80* expression in EpiWAT from SAKO (n=9) and Flox (n=9) mice. **b** Immunostaining for F4/80 in the EpiWAT of SAKO and Flox mice. Representative images from six independent experiments are shown, as well as the quantitative analysis results. ATM, adipose tissue macrophage. Scale bar, 20 μm. **c** qPCR analysis of the expression of the inflammatory factors *Il-6*, *Il-1β*, *Tnf-α*, and *Ccl2* in EpiWAT from SAKO (n=9) and Flox (n=9) mice. **d** Representative western blots for Il-6, Tnf-α, Ccl2 and β-Actin proteins in the EpiWAT of SAKO (n=3) and Flox (n=3) mice fed an NCD. Right panel, summary of the quantification of three independent experiments. **e** Weight of livers from SAKO (n=9) and Flox (n=9) mice. **f** HE staining results of liver tissues from SAKO and Flox mice. Representative images from three independent experiments are shown. Scale bar, 20 μm. **g** Hepatic TG, TC and FFA levels in SAKO (n=6) and Flox (n=6) mice. **h** Western blots for Slc35d3, Notch1, Hes1 and β-Actin protein in the livers of SAKO (n=3) and Flox (n=3) mice. Right panel, summary of the quantification of three independent experiments. Data are expressed as the mean ± SEM. Statistical significance was assessed by unpaired two-sided Student's t test (a-e and g-h). The exact *P* values are shown in the figure. SAKO, *Slc35d3* adipocyte-specific knockout; EpiWAT, epididymal white adipose tissue; TG, triglyceride; TC, total cholesterol; FFA, free fatty acid. Source data are provided as a Source Data file.

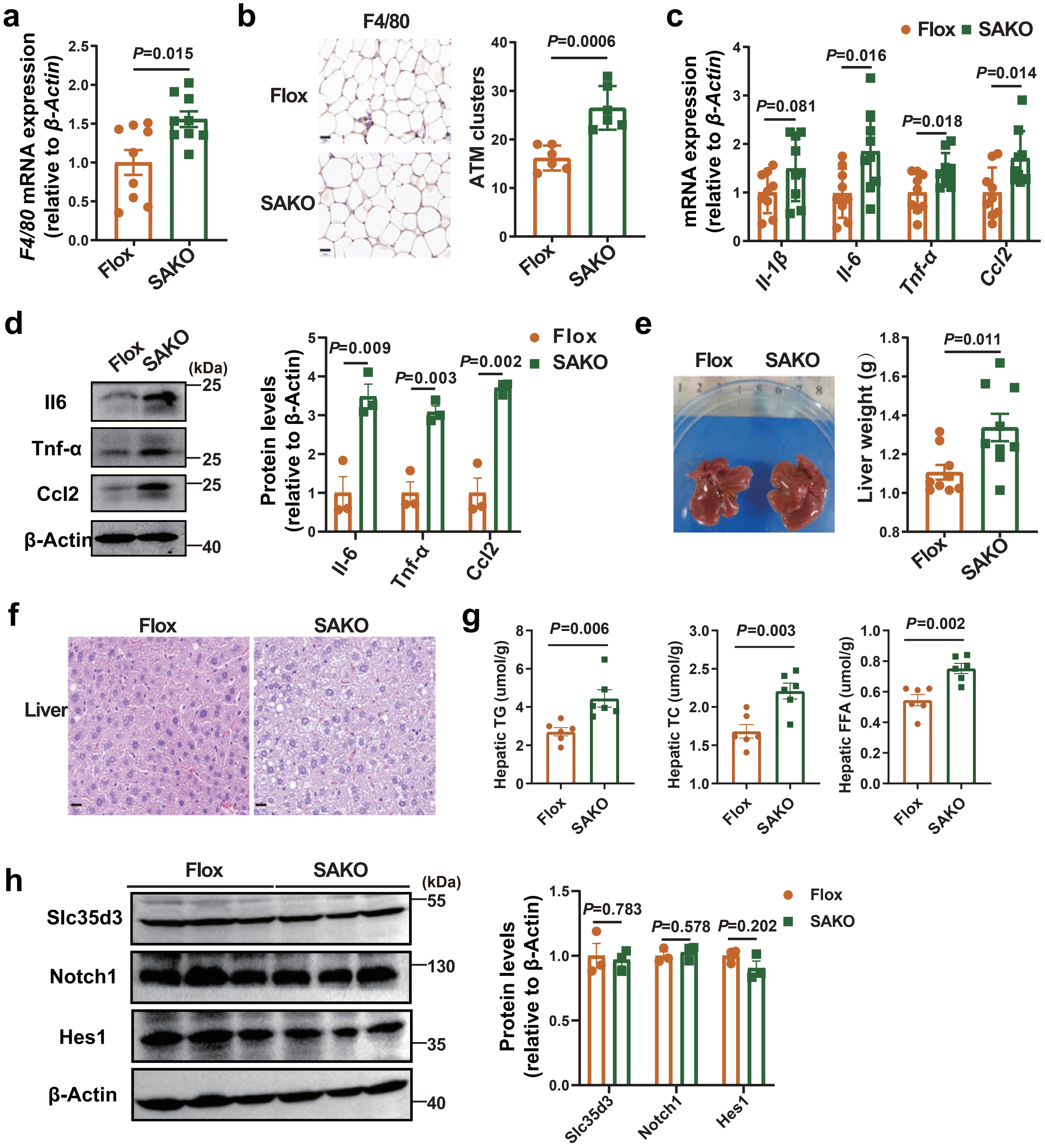
a
F4/80 mRNA expression (relative to β-Actin)
P=0.015
Flox
SAKO
b
F4/80
Flox
SAKO
ATM clusters
P=0.0006
c
Flox SAKO
mRNA expression (relative to β-Actin)
P=0.081
P=0.016
P=0.018
P=0.014
Il-1β
Il-6
Tnf-α
Ccl2
d
Flox SAKO
(kDa)
Il6
Tnf-α
Ccl2
β-Actin
25
25
25
40
Protein levels (relative to β-Actin)
P=0.009
P=0.003
P=0.002
Il-6
Tnf-α
Ccl2
e
Flox
SAKO
Liver weight (g)
P=0.011
f
Flox
SAKO
Liver
g
Hepatic TG (umol/g)
P=0.006
Hepatic TC (umol/g)
P=0.003
Hepatic FFA (umol/g)
P=0.002
h
Flox
SAKO
(kDa)
Slc35d3
Notch1
Hes1
β-Actin
55
130
35
40
Protein levels (relative to β-Actin)
P=0.783
P=0.578
P=0.202
Slc35d3
Notch1
Hes1

Supplementary Figure 2. Browning phenotypes of the EpiWAT and BAT in SAKO mice.

a, e HE staining results of the EpiWAT (a) and BAT (e) from SAKO and Flox mice. Representative HE images from three independent experiments are shown. Right panel of a, the quantitative results of the size of each adipocyte counted (Flox, n=113 cells; SAKO, n=102 cells). Scale bar, 50 μm. **b, f** qPCR analysis of *Ucp1* expression in the EpiWAT (b) and BAT (f) from SAKO (n=6) mice and their Flox littermates (n=6). **c, g** Immunostaining for Ucp1 in the EpiWAT (c) and BAT (g) from SAKO and Flox mice. Representative images from three independent experiments are shown. Scale bar, 50 μm. **d, h** Representative western blot results of Ucp1 expression in the EpiWAT (d) and BAT (h) from SAKO (n=3) mice and their Flox littermates (n=3). Right panel, summary of the quantification of three independent experiments. Data are expressed as the mean ± SEM. Statistical significance was assessed by unpaired two-sided Student's t test (a-b, d, f and h). The exact *P* values are shown in the figure. SAKO, *Slc35d3* adipocyte-specific knockout; EpiWAT, epididymal white adipose tissue; BAT, brown adipose tissue. Source data are provided as a Source Data file.

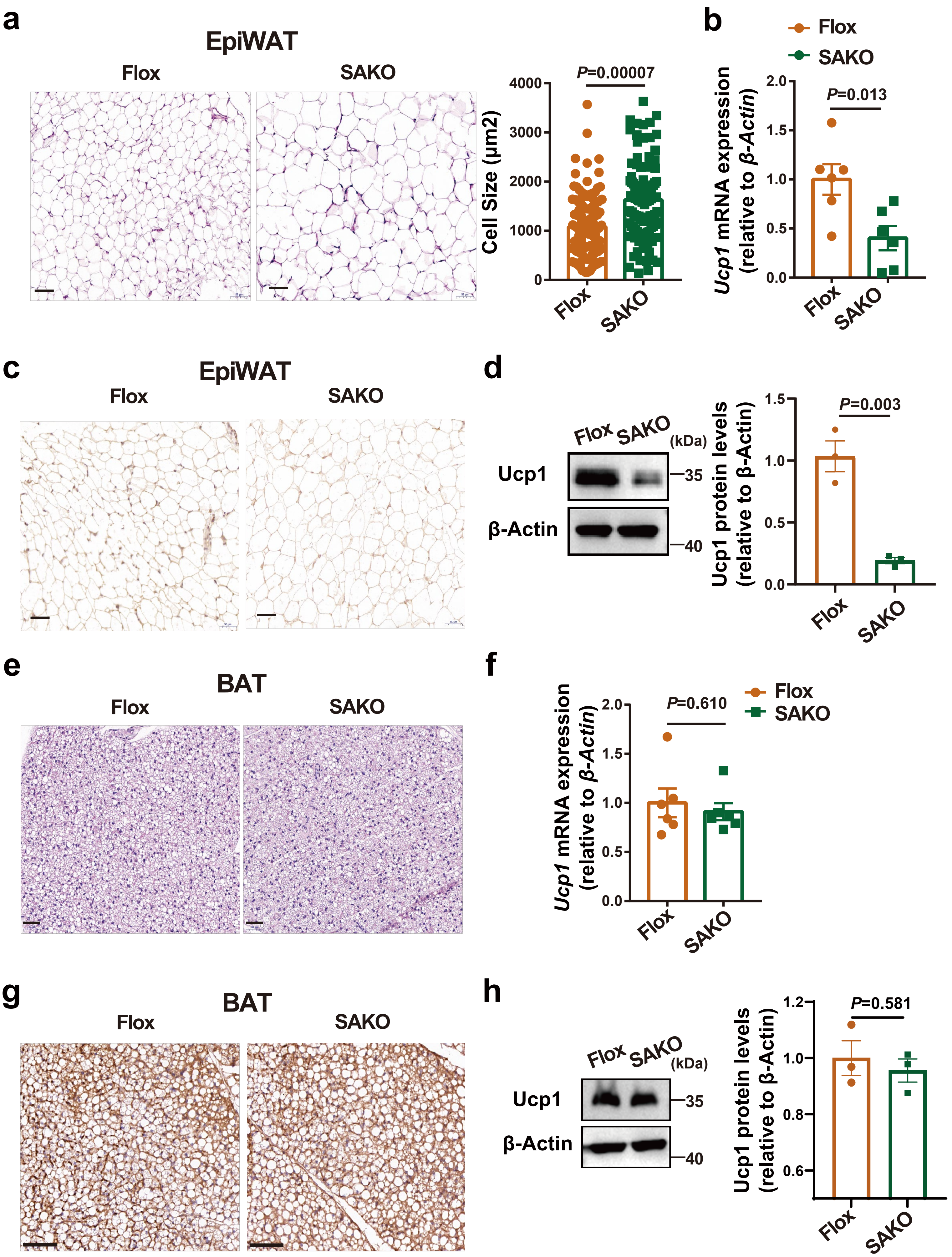
a
EpiWAT
Flox
SAKO
P=0.00007
Cell Size (μm2)
4000
3000
2000
1000
0
Flox
SAKO
b
Flox
SAKO
P=0.013
Ucp1 mRNA expression
(relative to β-Actin)
2.0
1.5
1.0
0.5
0.0
Flox
SAKO
c
EpiWAT
Flox
SAKO
d
Flox
SAKO
(kDa)
Ucp1
β-Actin
35
40
P=0.003
Ucp1 protein levels
(relative to β-Actin)
1.5
1.0
0.5
0.0
Flox
SAKO
e
BAT
Flox
SAKO
f
P=0.610
Flox
SAKO
Ucp1 mRNA expression
(relative to β-Actin)
2.0
1.5
1.0
0.5
0.0
Flox
SAKO
g
BAT
Flox
SAKO
h
Flox
SAKO
(kDa)
Ucp1
β-Actin
35
40
P=0.581
Ucp1 protein levels
(relative to β-Actin)
1.2
1.0
0.8
0.6
Flox
SAKO

Supplementary Figure 3. Adipose-specific *Slc35d3* knock-in protected mice from obesity.

a Schematic diagram of NCD feeding. SAKI and WT mice were fed an NCD from 4 to 18 weeks of age. **b** Body weight curve of SAKI (n=5) and WT (n=11) mice fed an NCD (*P*=0.047, 0.008, 0.009, 0.015, 0.026, 0.003, 0.004, 0.032 for SAKI mice and controls from 9 to 16 weeks). **c-e** Body weights (c), body lengths (d) and fat pad weights (e) of SAKI (n=6) mice and WT (n=6) mice fed an NCD. **f** Serum lipid profiles (TG, TC and FFA) in SAKI (n=9) mice and their WT littermates (n=9). **g-h** Blood glucose concentrations during IP-GTT (g) and IP-ITT (h) in NCD-fed SAKI mice and their WT littermates; n=5 per group. AUC values are shown. Data are expressed as the mean ± SEM. Statistical significance was assessed by unpaired two-sided Student's t test (c-f) or two-way ANOVA followed by Bonferroni's multiple comparisons test (b and g-h). The exact *P* values are shown in the figure or corresponding legends; **P*<0.05. AUC, area under the curve; EpiWAT, epididymal white adipose tissue; BAT, brown adipose tissue; IngWAT, inguinal white adipose tissue; AsWAT, anterior-subcutaneous white adipose tissue; TG, triglyceride; TC, total cholesterol; FFA, free fatty acid; NCD, normal chow diet; SAKI, *Slc35d3* adipocyte-specific knockin. Source data are provided as a Source Data file.

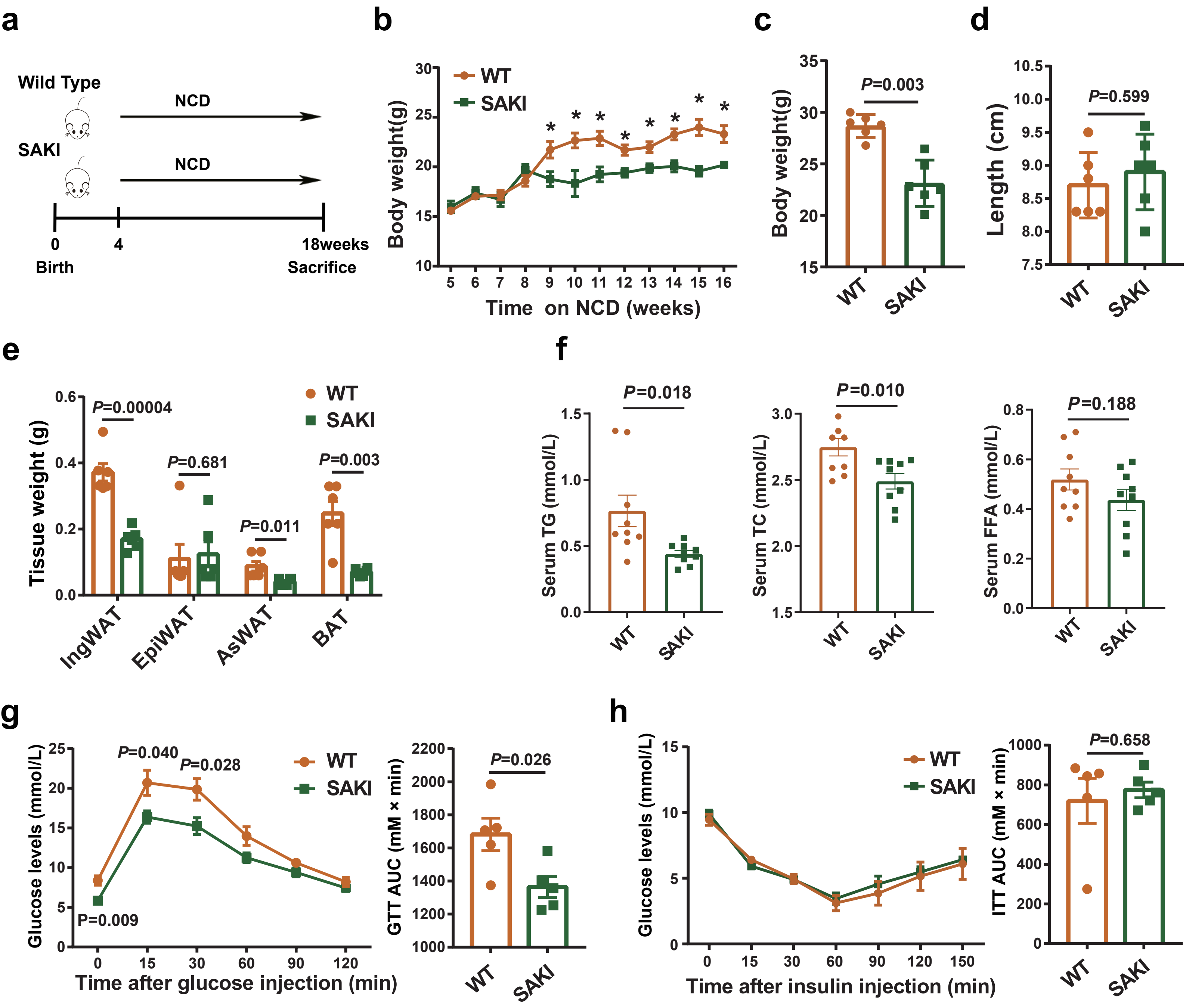
a
Wild Type
NCD
SAKI
NCD
0
4
18weeks
Birth
Sacrifice
b
Body weight(g)
WT
SAKI
Time on NCD (weeks)
c
Body weight(g)
P=0.003
WT
SAKI
d
Length (cm)
P=0.599
WT
SAKI
e
Tissue weight (g)
P=0.00004
P=0.681
P=0.011
P=0.003
WT
SAKI
IngWAT
EpiWAT
AsWAT
BAT
f
Serum TG (mmol/L)
P=0.018
Serum TC (mmol/L)
P=0.010
Serum FFA (mmol/L)
P=0.188
WT
SAKI
g
Glucose levels (mmol/L)
P=0.040
P=0.028
P=0.009
WT
SAKI
Time after glucose injection (min)
GTT AUC (mM × min)
P=0.026
h
Glucose levels (mmol/L)
WT
SAKI
Time after insulin injection (min)
ITT AUC (mM × min)
P=0.658

Supplementary Figure 4. Adipose-specific *Slc35d3* knockin protected mice from adipose tissue inflammation and liver steatosis.

a, i qPCR analysis results of macrophagocytic marker *F4/80* in the EpiWAT from SAKI and WT mice fed an NCD (a) or HFD (i); n=9 per group. **b, j** Immunostaining for F4/80 in the EpiWAT from SAKI and WT mice fed an NCD (b) or HFD (j). Representative images and quantitative analysis results of six independent experiments are shown. ATM, adipose tissue macrophage. Scale bar, 20 μm. **c, k** qPCR analysis results of the inflammatory factors *Il-6*, *Il-1β*, *Tnf-α*, and *Ccl2* in the EpiWAT from SAKI and WT mice fed an NCD (c) or HFD (k); n=9 per group. **d, l** Representative western blot for Il-6, Tnf-α, Ccl2 and β-Actin in the EpiWAT from SAKI and WT mice fed an NCD (d) or HFD (l); n=3 per group. The quantification of three independent experiments is shown in the right panel. **e, m** Weight of livers from SAKI and WT mice fed an NCD (e, n=9 per group) or HFD (m, n=6 per group). **f, n** HE staining results of liver tissues from SAKI and WT mice fed an NCD (f) or HFD (n). Representative images from three independent experiments are shown. Scale bar, 20 μm. **g, o** Hepatic TG, TC and FFA levels in SAKI and WT mice fed an NCD (g) or HFD (o); n=6 per group. **h, p** Western blots for Slc35d3, Notch1, Hes1 and β-Actin in the liver of SAKI and WT mice fed an NCD (h) or HFD (p); n=3 per group. The quantification of three independent experiments is shown in the right panel. Data are expressed as the mean ± SEM. Statistical significance was assessed by unpaired two-sided Student's t test (a-e, g-m, o-p). The exact *P* values are shown in the figure. EpiWAT, epididymal white adipose tissue; TG, triglyceride; TC, total cholesterol; FFA, free fatty acid; NCD, normal chow diet; HFD, high fat diet; SAKI, *Slc35d3* adipocyte-specific knockin. Source data are provided as a Source Data file.

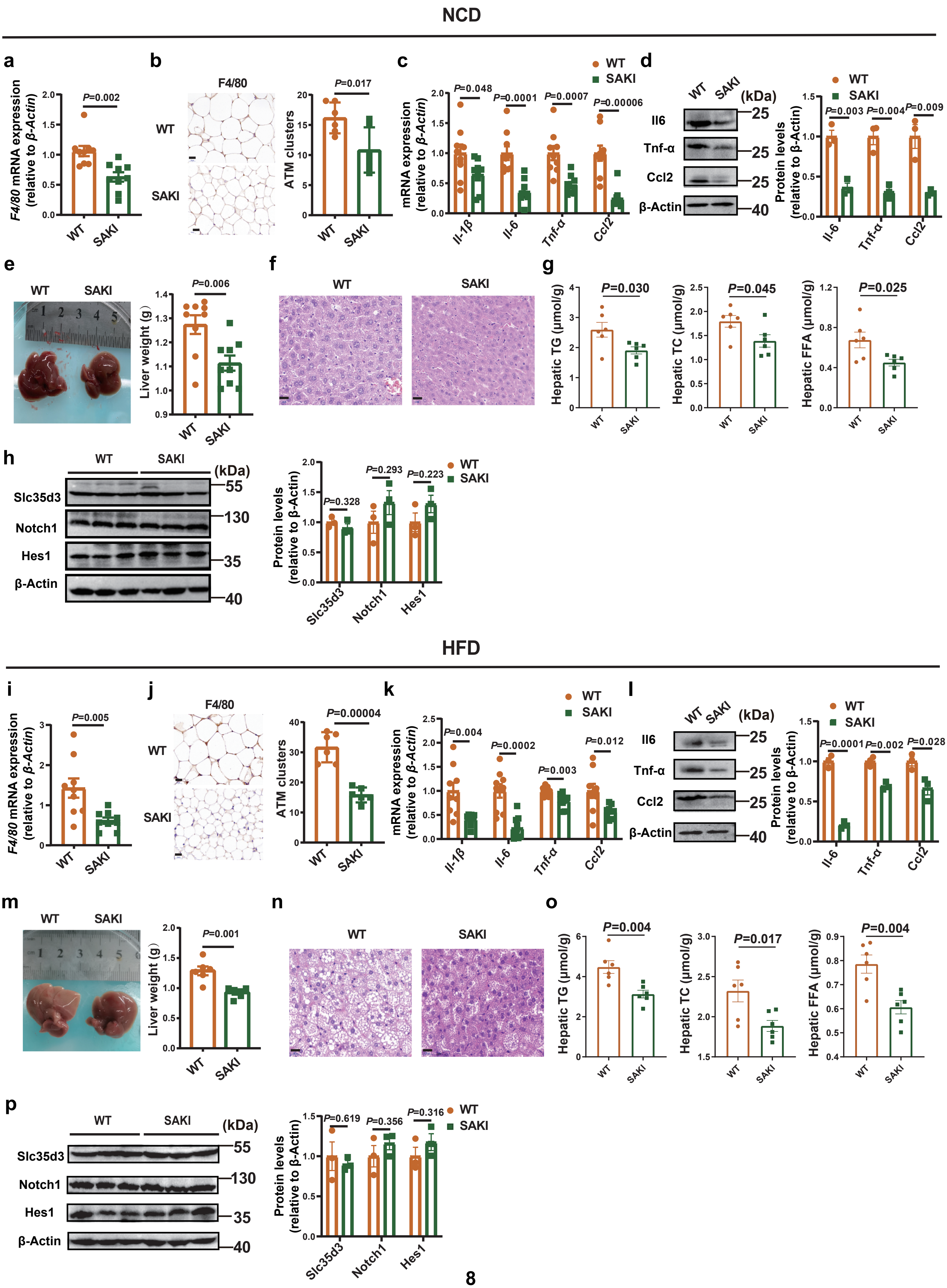
NCD
a
F4/80 mRNA expression (relative to β-Actin)
P=0.002
WT
SAKI
b
F4/80
ATM clusters
P=0.017
c
mRNA expression (relative to β-Actin)
P=0.048
P=0.0001
P=0.0007
P=0.00006
Il-1β
Il-6
Tnf-α
Ccl2
d
(kDa)
Il6
Tnf-α
Ccl2
β-Actin
25
40
Protein levels (relative to β-Actin)
P=0.003
P=0.004
P=0.009
e
Liver weight (g)
P=0.006
f
g
Hepatic TG (μmol/g)
P=0.030
Hepatic TC (μmol/g)
P=0.045
Hepatic FFA (μmol/g)
P=0.025
h
Slc35d3
Notch1
Hes1
55
130
35
P=0.328
P=0.293
P=0.223
HFD
i
P=0.005
j
P=0.00004
k
P=0.004
P=0.0002
P=0.003
P=0.012
l
P=0.0001
P=0.002
P=0.028
m
P=0.001
n
o
P=0.004
P=0.017
P=0.004
p
P=0.619
P=0.356
P=0.316

Supplementary Figure 5. Browning phenotype of IngWAT in NCD-fed SAKI mice.

a Food intake of NCD-fed SAKI (n=30) mice and WT mice (n=30). **b** Activity of NCD-fed SAKI (n=5) mice and WT mice (n=5). **c-e** Oxygen consumption (c), carbon dioxide production (d) and energy expenditure (e) of NCD-fed SAKI (n=5) and WT mice (n=5). **f** The various components of oxygen consumption rates of mature adipocytes from NCD-fed SAKI (n=3) and WT (n=3) mice. The asterisks in the figure represent the exact *P*-values in the order: *P*=0.033, *P*=0.029, *P*=0.041, *P*=0.006, *P*=0.012. **g** Rectal temperatures of NCD-fed SAKI (n=5) mice and WT mice (n=5), which were recorded during cold stimulation for 4 h and returned to RT for 10 min. **h-j** The expression levels of thermogenesis genes (h), beige adipocyte markers (i), and mitochondria-related genes (j) in the IngWAT of NCD-fed SAKI (n=9) and WT mice (n=9). **k** Representative western blots of thermogenesis proteins in the IngWAT from SAKI and WT mice; n=3 per group. The quantification of three independent experiments is shown. **l** Immunostaining for Ucp1 in the IngWAT of NCD-fed SAKI mice and WT mice. Representative images from three independent experiments are shown. Scale bar, 50 μm. **m** HE staining and adipocyte cell size measurements of IngWAT from NCD-fed SAKI mice and WT mice. Representative HE images from three independent experiments are shown. Right panel, the quantitative results of the size of each adipocyte counted (WT, n=110 cells; SAKI, n=106 cells). Scale bar, 500 μm (left); 20 μm (right). **n** Expression levels of lipolysis genes in the IngWAT from NCD-fed SAKI (n=9) mice and WT mice (n=9). Data are expressed as the mean ± SEM. Statistical significance was assessed by unpaired two-sided Student's t test (a-b, f-k and m-n) or two-way ANCOVA (c-e). The exact *P* values are shown in the figure or corresponding legends; *$P<0.05$. SAKI, *Slc35d3* adipocyte-specific knockin; IngWAT, inguinal white adipose tissue; RT, room temperature. Source data are provided as a Source Data file.

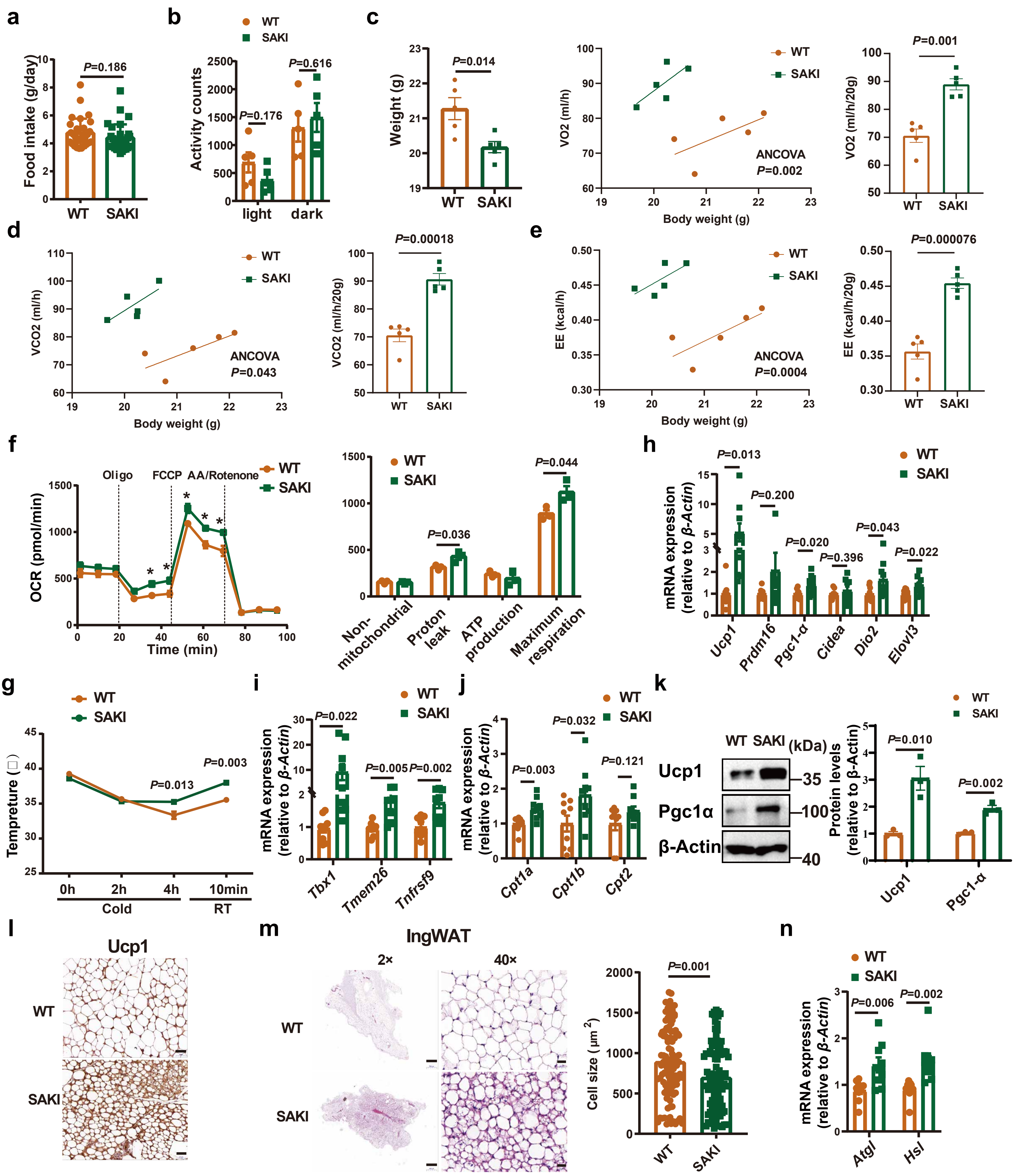
a
Food intake (g/day)
P=0.186
WT
SAKI
b
WT
SAKI
Activity counts
P=0.176
P=0.616
light
dark
c
Weight (g)
P=0.014
VO2 (ml/h)
Body weight (g)
ANCOVA
P=0.002
VO2 (ml/h/20g)
P=0.001
d
VCO2 (ml/h)
ANCOVA
P=0.043
VCO2 (ml/h/20g)
P=0.00018
e
EE (kcal/h)
ANCOVA
P=0.0004
EE (kcal/h/20g)
P=0.000076
f
Oligo
FCCP
AA/Rotenone
OCR (pmol/min)
Time (min)
P=0.036
P=0.044
Non-mitochondrial
Proton leak
ATP production
Maximum respiration
h
mRNA expression (relative to β-Actin)
P=0.013
P=0.200
P=0.020
P=0.396
P=0.043
P=0.022
Ucp1
Prdm16
Pgc1-α
Cidea
Dio2
Elovl3
g
Tempreture (□)
P=0.013
P=0.003
0h
2h
4h
10min
Cold
RT
i
P=0.022
P=0.005
P=0.002
Tbx1
Tmem26
Tnfrsf9
j
P=0.003
P=0.032
P=0.121
Cpt1a
Cpt1b
Cpt2
k
(kDa)
Ucp1
Pgc1α
β-Actin
35
100
40
Protein levels (relative to β-Actin)
P=0.010
P=0.002
Pgc1-α
l
Ucp1
m
IngWAT
2×
40×
Cell size (μm²)
P=0.001
n
P=0.006
P=0.002
Atgl
Hsl

Supplementary Figure 6. Adipocyte size of EpiWAT in SAKI mice.

a HE staining and adipocyte cell size measurements of EpiWAT in NCD-fed SAKI mice and their WT littermates. Representative HE images from three independent experiments are shown. Right panel, the quantitative results of the size of each adipocyte counted (WT, n=117 cells; SAKI, n=140 cells). Scale bar, 50 μm. **b** HE staining and adipocyte cell size measurements of EpiWAT in HFD-fed SAKI mice and their WT littermates. Representative HE images from three independent experiments are shown. Right panel, the quantitative results of the size of each adipocyte counted (WT, n=100 cells; SAKI, n=126 cells). Scale bar, 50 μm. Data are expressed as the mean ± SEM. Statistical significance was assessed by unpaired two-sided Student's t test (a-b). The exact *P* values are shown in the figure. EpiWAT, epididymal white adipose tissue; NCD, normal chow diet; HFD, high fat diet; SAKI, *Slc35d3* adipocyte-specific knockin. Source data are provided as a Source Data file.

NCD

a

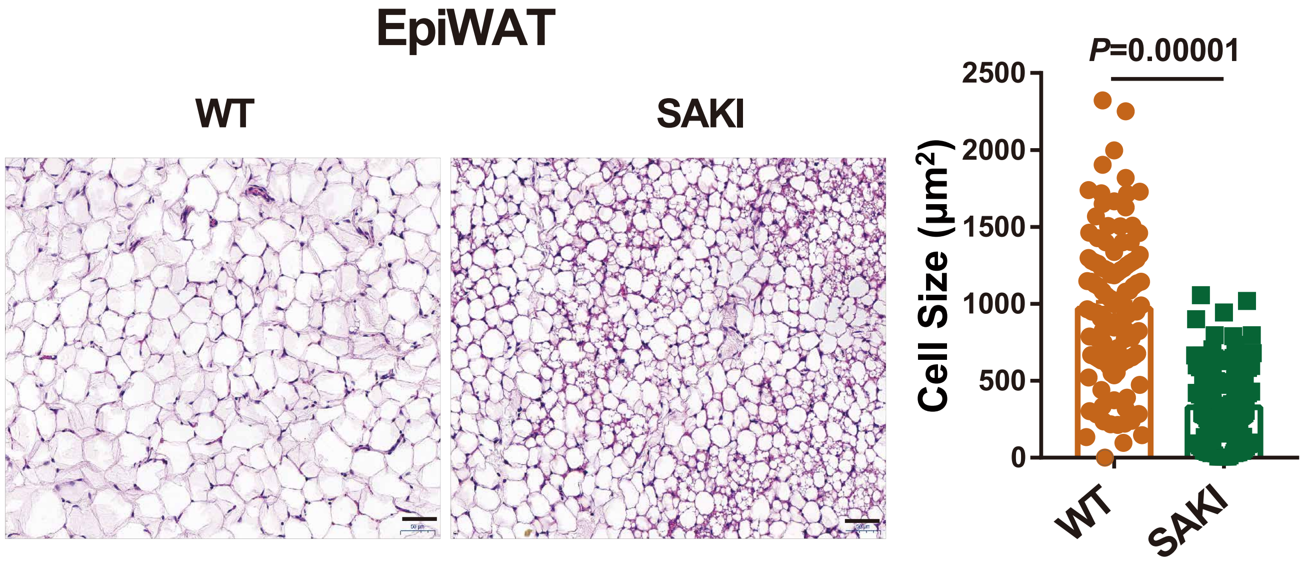
EpiWAT
WT
SAKI
P=0.00001
2500
2000
1500
1000
500
0
Cell Size (μm²)
WT
SAKI

HFD

b

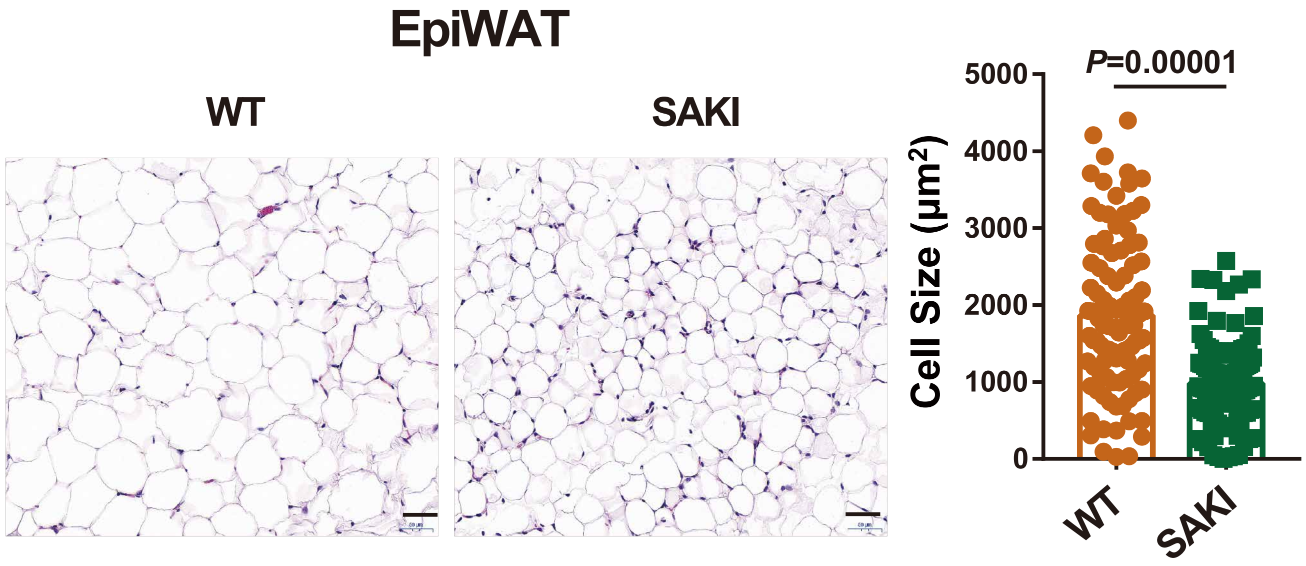
EpiWAT
WT
SAKI
P=0.00001
5000
4000
3000
2000
1000
0
Cell Size (μm²)
WT
SAKI

Supplementary Figure 7. The interaction between SLC35D3 and NOTCH1. **a** In SLC35D3-overexpressing cells, SLC35D3 and NOTCH1 colocalized. Representative confocal images of three independent experiments are shown, and the inset in the bottom panel shows a magnified view of the indicated area. Scale bar, 20 μm. **b** CoIP assay results showed no interaction between SLC35D3 and NICD. Representative images from three independent experiments are shown. **c** CoIP assay results showed the interaction between SLC35D3-30aa and ECD. Representative images from three independent experiments are shown. **d-e** Multiplex fluorescence staining results of cells transfected with SLC35D3 plasmid and ECD plasmid, as well as cells transfected with SLC35D3 plasmid and NICD plasmid. SLC35D3 and ECD colocalized and both colocalized with ER (d), while SLC35D3 and NICD did not colocalize (e). Representative confocal images of four independent experiments are shown, and the inset in the bottom panel shows a magnified view of the indicated area. Scale bar, 50 μm. **f-g** NOTCH1 levels were downregulated in the plasma membrane when SLC35D3 was overexpressed (f). The quantitative analysis results of three independent experiments (g) are shown. **h-i** Membrane NOTCH1 levels in SLC35D3-overexpressing HEK293T cells and control cells were detected by flow cytometric analysis. Binding curves were generated by determining the NOTCH1 mean fluorescence density (MFI). The quantitative analysis results of three independent experiments (i) are shown. **j** Live cell imaging results obtained using a high-content imaging system. Representative images of four independent experiments are shown, and the inset in the right panel shows a magnified view of the indicated area. Scale bar, 20 μm. Data are expressed as the mean ± SEM. Statistical significance was assessed by unpaired two-sided Student's t test (g, i). The exact P values are shown in the figure. OE, overexpression; ER, endoplasmic reticulum. Source data are provided as a Source Data file.

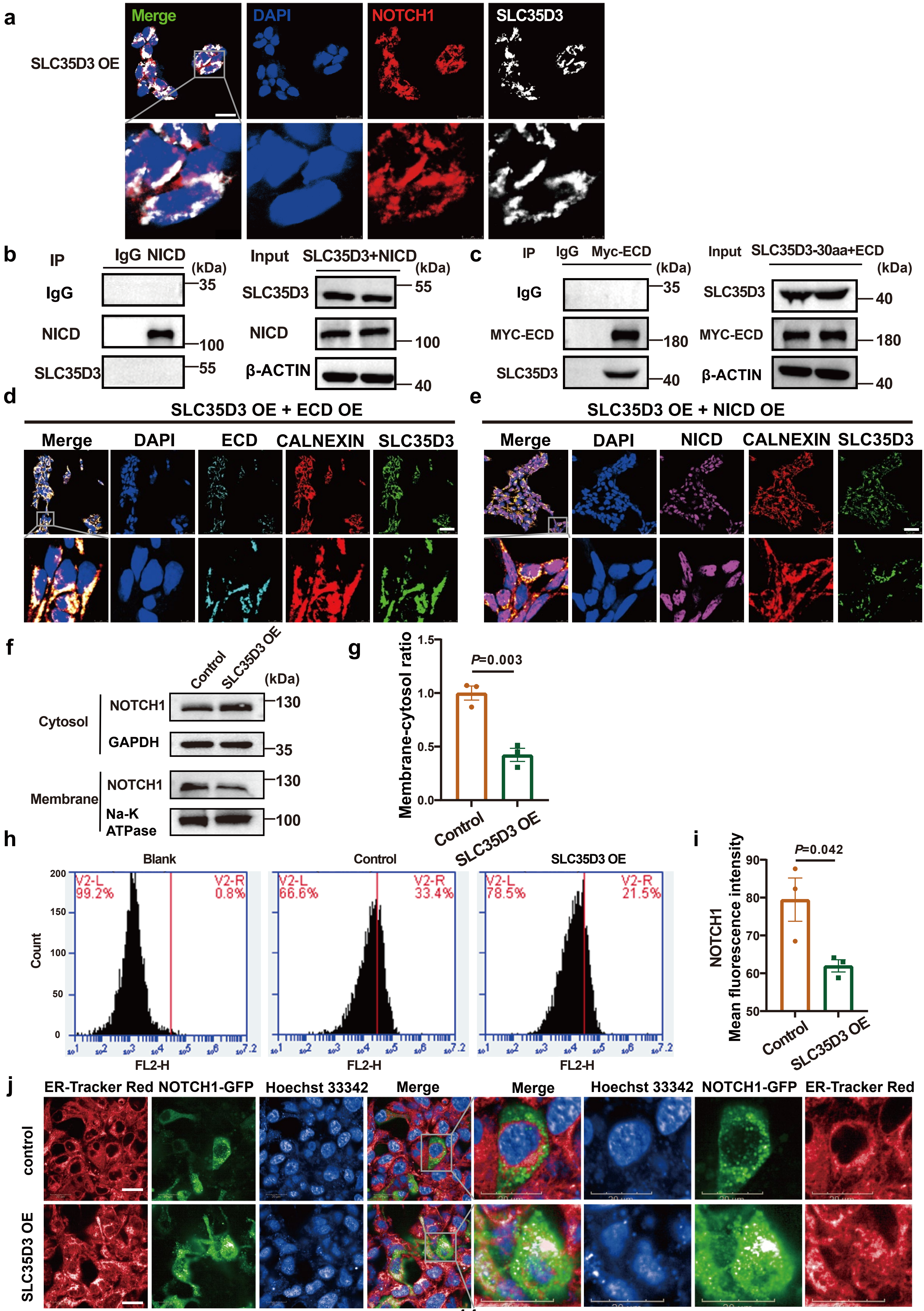
a
Merge
DAPI
NOTCH1
SLC35D3
SLC35D3 OE
b
IP
IgG NICD
(kDa)
IgG
NICD
SLC35D3
35
100
55
Input
SLC35D3+NICD
SLC35D3
NICD
β-ACTIN
55
100
40
c
IP
IgG
Myc-ECD
(kDa)
IgG
MYC-ECD
SLC35D3
35
180
40
Input
SLC35D3-30aa+ECD
SLC35D3
MYC-ECD
β-ACTIN
40
180
40
d
SLC35D3 OE + ECD OE
Merge
DAPI
ECD
CALNEXIN
SLC35D3
e
SLC35D3 OE + NICD OE
Merge
DAPI
NICD
CALNEXIN
SLC35D3
f
Control
SLC35D3 OE
(kDa)
Cytosol
NOTCH1
GAPDH
130
35
Membrane
NOTCH1
Na-K ATPase
130
100
g
Membrane-cytosol ratio
P=0.003
Control
SLC35D3 OE
h
Blank
Control
SLC35D3 OE
V2-L 99.2%
V2-R 0.8%
V2-L 66.6%
V2-R 33.4%
V2-L 78.5%
V2-R 21.5%
Count
FL2-H
i
NOTCH1 Mean fluorescence intensity
P=0.042
Control
SLC35D3 OE
j
ER-Tracker Red
NOTCH1-GFP
Hoechst 33342
Merge
Merge
Hoechst 33342
NOTCH1-GFP
ER-Tracker Red
control
SLC35D3 OE

Supplementary Figure 8. SLC35D3 regulated NOTCH1 export from the ER. **a-b** SLC35D3 knockdown reduced the colocalization of NOTCH1 and ER. Representative confocal images of cells are shown (a), as well as the quantification results of four independent parallel experiments (b). The inset in the right panel shows a magnified view of the indicated area. Scale bar, 20 μm. CALNEXIN, an ER marker protein. **c-d** SAKI mice showed lower levels of Notch1 at the cell membrane in IngWAT than control mice. Representative confocal images are shown (c), as well as the quantification results of six independent parallel experiments (d). Scale bar, 50 μm. Na-K ATPase, a plasma membrane marker protein. Data are expressed as the mean ± SEM. Statistical significance was assessed by unpaired two-sided Student's t test (b, d). The exact P values are shown in the figure. ER, endoplasmic reticulum; IngWAT, inguinal white adipose tissue; SAKI, *Slc35d3* adipocyte-specific knockin. Source data are provided as a Source Data file.

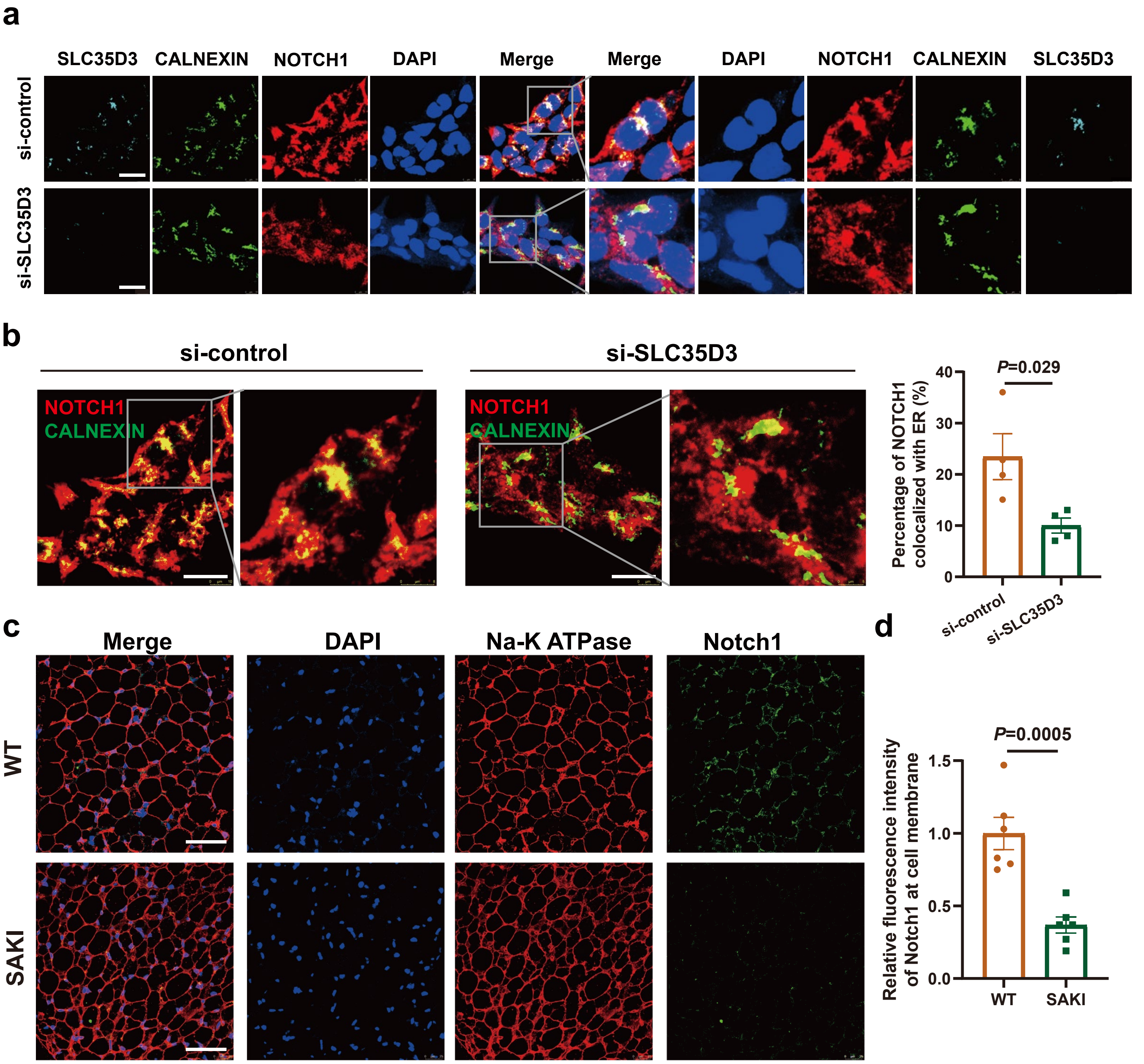
a
SLC35D3
CALNEXIN
NOTCH1
DAPI
Merge
Merge
DAPI
NOTCH1
CALNEXIN
SLC35D3
si-control
si-SLC35D3
b
si-control
si-SLC35D3
NOTCH1
CALNEXIN
NOTCH1
CALNEXIN
P=0.029
Percentage of NOTCH1 colocalized with ER (%)
40
30
20
10
0
si-control
si-SLC35D3
c
Merge
DAPI
Na-K ATPase
Notch1
WT
SAKI
d
P=0.0005
Relative fluorescence intensity of Notch1 at cell membrane
1.5
1.0
0.5
0.0
WT
SAKI

Supplementary Figure 9. SLC35D3 regulated NOTCH1 levels in the ER and cell membrane.

a-d Representative western blotting results (a) of total NOTCH1, membrane NOTCH1, and ER NOTCH1 proteins at different time points in control and SLC35D3 overexpressing cells. The quantitative analysis results of three independent experiments (b-d) are shown. **e-h** Representative western blotting results (e) of total NOTCH1, membrane NOTCH1, and ER NOTCH1 proteins at different time points in control and SLC35D3 knockdown cells. The quantitative analysis results of three independent experiments (f-h) are shown. **i-j** Representative western blotting results (i) of total NOTCH1, membrane NOTCH1, and ER NOTCH1 proteins 48 h after transfection (i). The quantitative analysis results of NOTCH1 levels at 72 h in three independent experiments (j) are shown. **k** Western blotting results of cell surface biotinylation assays in 293T cells and primary adipocytes; PBS incubation served as a negative control. Representative images from three independent experiments are shown. **l-n** 293T cells were transfected with SLC35D3 plasmid and siRNA, and then cells were harvested 48 h and 72 h after transfection for cell surface biotinylation assays. Representative blots (l) from three independent experiments are shown, as well as the quantitative analysis results of three independent experiments (m-n). Total NOTCH1 expression was normalized to β-ACTIN expression, membrane NOTCH1 expression was normalized to Na-K ATPase, and ER NOTCH1 expression was normalized to CALNEXIN (a-j). Data are expressed as the mean ± SEM. Statistical significance was assessed by unpaired two-sided Student's t test (b-d, f-h, j, m-n). The exact P values are shown in the figure. OE, overexpression; ER, endoplasmic reticulum. Source data are provided as a Source Data file.

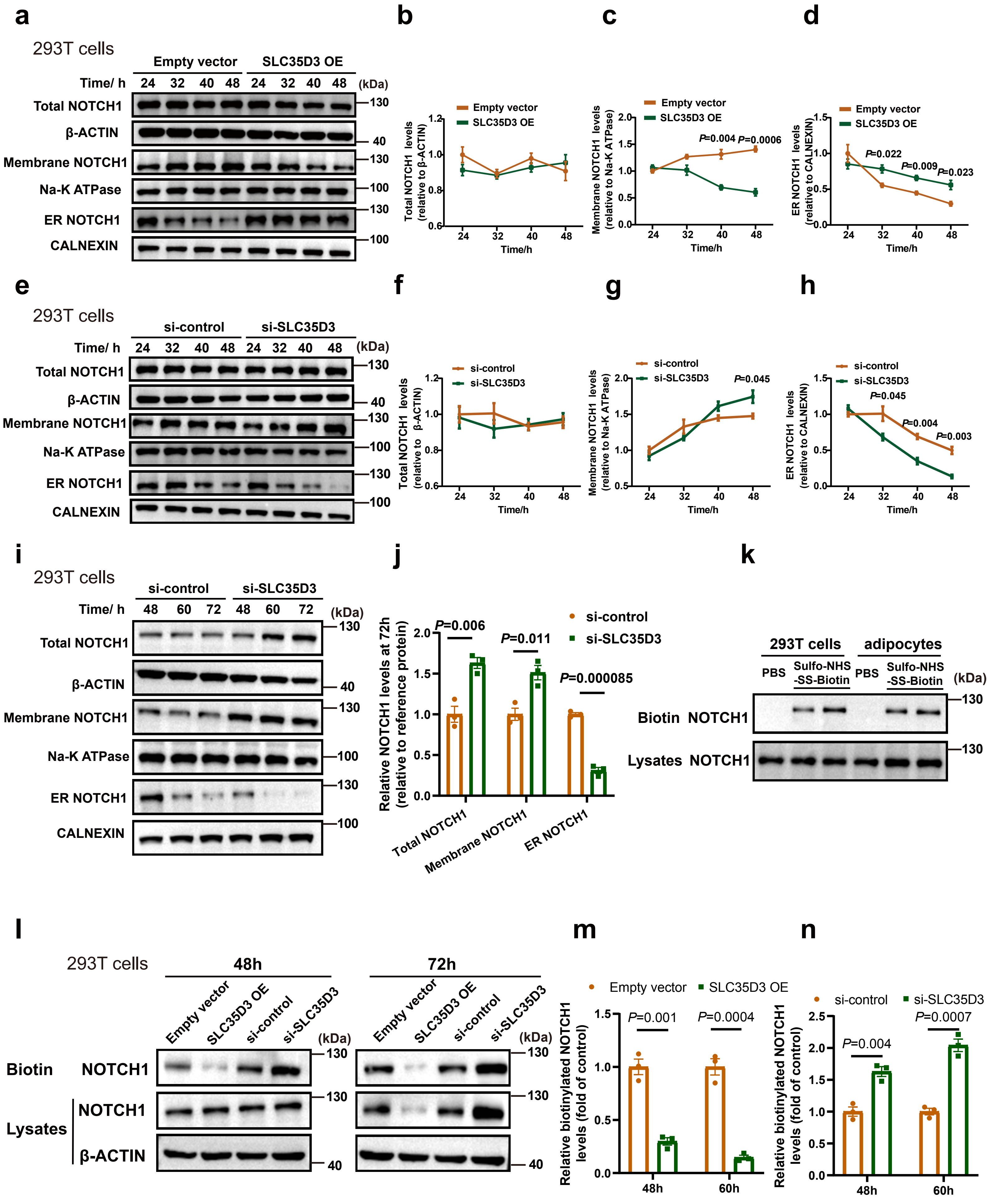

a
293T cells
Empty vector
SLC35D3 OE
Time/ h
24 32 40 48 24 32 40 48
(kDa)
Total NOTCH1
β-ACTIN
Membrane NOTCH1
Na-K ATPase
ER NOTCH1
CALNEXIN
130
40
130
100
130
100
b
Empty vector
SLC35D3 OE
Total NOTCH1 levels (relative to β-ACTIN)
Time/h
c
Membrane NOTCH1 levels (relative to Na-K ATPase)
P=0.004 P=0.0006
d
ER NOTCH1 levels (relative to CALNEXIN)
P=0.022
P=0.009
P=0.023
e
293T cells
si-control
si-SLC35D3
f
g
P=0.045
h
P=0.045
P=0.004
P=0.003
i
293T cells
Time/ h
48 60 72 48 60 72
j
si-control
si-SLC35D3
Relative NOTCH1 levels at 72h (relative to reference protein)
P=0.006
P=0.011
P=0.000085
Total NOTCH1
Membrane NOTCH1
ER NOTCH1
k
293T cells
adipocytes
PBS
Sulfo-NHS -SS-Biotin
Biotin NOTCH1
Lysates NOTCH1
l
293T cells
48h
72h
Empty vector
SLC35D3 OE
si-control
si-SLC35D3
Biotin
NOTCH1
Lysates
β-ACTIN
m
Empty vector
SLC35D3 OE
Relative biotinylated NOTCH1 levels (fold of control)
P=0.001
P=0.0004
48h
60h
n
si-control
si-SLC35D3
P=0.004
P=0.0007

Supplementary Table 1. Antibodies and dilutions

Antibodies	Brands	Dilutions
Anti-SLC35D3	Thermo scientific, PA572721	1:500, 1:1000
Anti-Myc	Abcam, ab206486; CST, 2276S	1:50, 1:1000
Anti-his	CST, 12698S	1:50, 1:1000
Anti-Hes1	Santa, sc-166410	1:1000
Anti-Notch1	CST, 3608S; 4380S	1:200, 1:1000
Anti-Nicd	CST, 4147S	1:200, 1:1000
Anti-Ucp1	Abcam, ab10983	1:500, 1:1000
Anti-PGC1α	Proteintech, 66369-1-Ig	1:5000
Anti-Prdm16	Abcam, ab303534	1:1000
Anti-Na/K ATPase	Abcam, ab76020; ab7671	1:500, 1:1000
Anti-Calnexin	Thermo scientific, MA3027	1:500, 1:1000
Anti-Tnf-α	Proteintech, 60291-1-Ig	1:1000
Anti-Ccl2	Proteintech, 66272-1-Ig	1:1000
Anti-Il6	Huabio, EM1701-45	1:1000
Anti-F4/80	Proteintech, 28463-1-AP	1:2000
Anti-Gapdh	Proteintech, 60004-1-Ig	1:10000
PE-conjugated Notch1	CST, 15004S	1:50
Alexa Fluor™ 594	Thermo scientific, PA-11012, 11005	1:500
Alexa Fluor™ 488	Thermo scientific, PA-11034, 11029	1:500
HRP-conjugated Anti-ACTB	Proteintech, HRP-60008	1:2000

Anti-Rabbit IgG	Proteintech, SA00001-2	1:5000
Anti-Mouse IgG	Proteintech, SA00001-1	1:5000
Anti-Rat IgG	Proteintech, SA00001-15	1:5000

Supplementary Table 2. Primer sequences

Primer	Sequence
m*ACTB*-F	5'-GGCTGTATTCCCCTCCATCG-3'
m*ACTB*-R	5'-CCAGTTGGTAACAATGCCATGT-3'
m*Slc35d3*-F	5'-GTATGTGATCGCCGTCTCCG-3'
m*Slc35d3*-R	5'-CCGATCAGGATACAGGCCAC-3'
m*Notch1*-F	5'-GATGGCCTCAATGGGTACAAG-3'
m*Notch1*-R	5'-TCGTTGTTGTTGATGTCACAGT-3'
m*Hes1*-F	5'-TCAACACGACACCGGACAAAC-3'
m*Hes1*-R	5'-ATGCCGGGAGCTATCTTTCTT-3'
m*Hey1*-F	5'-CCGACGAGACCGAATCAATAAC-3'
m*Hey1*-R	5'-TCAGGTGATCCACAGTCATCTG-3'
m*Atgl*-F	5'-TCCGTGGCTGTCTACTAAAGA-3'
m*Atgl*-R	5'-TGGGATATGATGACGTTCTCTCC-3'
m*Hsl*-F	5'-GATTTACGCACGATGACACAGT-3'
m*Hsl*-R	5'-ACCTGCAAAGACATTAGACAGC-3'
m*Cpt1a*-F	5'-AGATCAATCGGACCCTAGACAC-3'
m*Cpt1a*-R	5'-CAGCGAGTAGCGCATAGTCA-3'
m*Cpt1b*-F	5'-TCTTCTTCCGACAAACCCTGA-3'
m*Cpt1b*-R	5'-GAGACGGACACAGATAGCCC-3'
m*Cpt2*-F	5'-CAAAAGACTCATCCGCTTTGTTC-3'
m*Cpt2*-R	5'-CATCACGACTGGGTTTGGGTA-3'

m*F4/80*-F	5’-AGGGTATCATGAGTTGATGGCA-3’
m*F4/80*-R	5’-TGTACCGTTGAAATAGGACGTG-3’
m*IL-6*-F	5’-TGATGGATGCTACCAAACTGGA-3’
m*IL-6*-R	5’-TGTGACTCCAGCTTATCTCTTGG-3’
m*IL-1β*-F	5’-TGCCACCTTTTGACAGTGATG-3’
m*IL-1β*-R	5’-AAGGTCCACGGGAAAGACAC-3’
m*Tnf-α*-F	5’-GTAGCCCACGTCGTAGCAA-3’
m*Tnf-α*-R	5’-TAGCAAATCGGCTGACGGTG-3’
m*Ccl2*-F	5’-AGATGCAGTTAACGCCCCAC-3’
m*Ccl2*-R	5’-CCCATTCCTTCTTGGGGTCA-3’
m*Ucp1*-F	5'-CAAAAACAGAAGGATTGCCGAAA-3'
m*Ucp1*-R	5'-TCTTGGACTGAGTCGTAGAGG-3'
m*Prdm16*-F	5'-CCCCACATTCCGCTGTGAT-3'
m*Prdm16*-R	5'-CTCGCAATCCTTGCACTCA-3'
mPgc1α-F	5'-TCTGAGTCTGTATGGAGTGACAT-3'
mPgc1α-R	5'-CCAAGTCGTTCACATCTAGTTCA-3'
m*Tbx1*-F	5'-GTCAAGGCTCCGGTGAAGAAG-3'
m*Tbx1*-R	5'-GCTGATTGAACTCGTCCCACA-3'
m*Tmem26*-F	5'-TTCTCCGGCCATCTTTGTGTA-3'
m*Tmem26*-R	5'-GTGCTGCAATACTGGTTTCCA-3'
m*Tnfrsf9*-F	5'-GTGCATTGAAGGATTCCATTGC-3'
m*Tnfrsf9*-R	5'-GCCAGTACCGTTCTGGTCATTA-3'

m*Shox2*-F	5'-CAAAGACGATGCGAAAGGGAT-3'
m*Shox2*-R	5'-AGGGTAAAATTGGTCCGACTTC-3'
m*ROSA26*-F	5'-GTATGTGATCGCCGTCTCCG-3'
m*ROSA26*-R	5'-TGAGCATGTCTTTAATCTACCTCGATG-3'
m*ROSA26*-R1	5'-GTCAATGGAAAGTCCCTATTGGCGT-3'
m*loxP*-F	5'-GCCTGAGCAACCTGGGCTCAGTAC-3'
m*loxP*-R	5'-AGAAACCCTCCTTGCCTCCATCTC-3'
m*Adiponectin*-F	5'-GGATGTGCCATGTGAGTCTG-3'
m*Adiponectin*-R	5'-ACGGACAGAAGCATTTTCCA-3'
