# Supplementary material for: SLC35D3 promotes white adipose tissue browning to ameliorate obesity by NOTCH signaling
Source: Nat Commun. 2023 Nov 23;14:7643. doi: 10.1038/s41467-023-43418-5 (PMC10667520; doi:10.1038/s41467-023-43418-5)
Supplement: Supplementary file 3 — Reporting Summary [file 41467_2023_43418_MOESM3_ESM.pdf]

nature portfolio

Corresponding author(s): Yibo Wang

Last updated by author(s): Nov 6, 2023

Reporting Summary

Nature Portfolio wishes to improve the reproducibility of the work that we publish. This form provides structure for consistency and transparency in reporting. For further information on Nature Portfolio policies, see our Editorial Policies and the Editorial Policy Checklist.

Statistics

For all statistical analyses, confirm that the following items are present in the figure legend, table legend, main text, or Methods section.

n/a	Confirmed	
☐	☒	The exact sample size (*n*) for each experimental group/condition, given as a discrete number and unit of measurement
☐	☒	A statement on whether measurements were taken from distinct samples or whether the same sample was measured repeatedly
☐	☒	The statistical test(s) used AND whether they are one- or two-sided<br>*Only common tests should be described solely by name; describe more complex techniques in the Methods section.*
☐	☒	A description of all covariates tested
☐	☒	A description of any assumptions or corrections, such as tests of normality and adjustment for multiple comparisons
☐	☒	A full description of the statistical parameters including central tendency (e.g. means) or other basic estimates (e.g. regression coefficient) AND variation (e.g. standard deviation) or associated estimates of uncertainty (e.g. confidence intervals)
☐	☒	For null hypothesis testing, the test statistic (e.g. *F*, *t*, *r*) with confidence intervals, effect sizes, degrees of freedom and *P* value noted<br>*Give P values as exact values whenever suitable.*
☒	☐	For Bayesian analysis, information on the choice of priors and Markov chain Monte Carlo settings
☒	☐	For hierarchical and complex designs, identification of the appropriate level for tests and full reporting of outcomes
☒	☐	Estimates of effect sizes (e.g. Cohen's *d*, Pearson's *r*), indicating how they were calculated

Our web collection on statistics for biologists contains articles on many of the points above.

Software and code

Policy information about availability of computer code

Data collection	The following tools were used for data collection:<br>-Indirect calorimetry system (Oxylet, PanLab, Spain)<br>-Copper thermocouple probe (FT3400 Type)<br>-XF extracellular flux analyzer (Seahorse Biosciences)<br>-Spectrophotometer (Nanodrop 2000c, Thermo Fisher)<br>-Prism 7500 sequence-detection system (ABI, Rockford, IL)<br>-Leica DM6000B with a digital camera (Leica, NY)<br>-High Content Imaging System (Opera Phenix, PerkinElmer)<br>-Leica SP8 Laser Confocal Microscope (Germany)<br>-Chemiluminescence system (Tanon 5800 Multi, China)<br>-Accu-Chek Active Blood Glucose Meter (Roche)<br>-Microplate reader (Infinite-M200)<br>-BD FACS-Calibur flow cytometer (BD Accuri C6)
Data analysis	The following software were used for data analysis:<br>-Image J software (version 2.0.0) was used for quantitative analysis.<br>-FlowJo CE (7.5.110.7) was used for analysis of FACS-Calibur data.<br>-GraphPad Prism (version 9.0) and SPSS (version 26.0) were used for statistical analysis.<br>-R (version 4.0.4), R packages fgsea (version 1.26.0) and clusterProfiler (version 4.8.2) were used for gene set enrichment analysis.

All the replicate experiments (including cell and mouse-based experiments) were biological replicates, which were repeated at least three times. Data are presented as means ± SEM and individual data points are plotted. The normality of the data was tested using the Shapiro–Wilk normality test. The differences between two groups were evaluated using unpaired two-sided Student's t tests, and multiple group comparisons were conducted by two-way ANOVA followed by Bonferroni's multiple comparisons test. A P value < 0.05 was considered statistically significant. Statistical analyses were performed in GraphPad PRISM 9 and SPSS 26.0. The images were created by Adobe Illustrator 2020 software.

For manuscripts utilizing custom algorithms or software that are central to the research but not yet described in published literature, software must be made available to editors and reviewers. We strongly encourage code deposition in a community repository (e.g. GitHub). See the Nature Portfolio guidelines for submitting code & software for further information.

Data

Policy information about availability of data

All manuscripts must include a data availability statement. This statement should provide the following information, where applicable:

- Accession codes, unique identifiers, or web links for publicly available datasets
- A description of any restrictions on data availability
- For clinical datasets or third party data, please ensure that the statement adheres to our policy

The authors declare that the data supporting the findings of this study are available within the manuscript, its supplementary information and the Source Data file. All data are publicly accessible. Source data are provided with this paper.

Research involving human participants, their data, or biological material

Policy information about studies with human participants or human data. See also policy information about sex, gender (identity/presentation), and sexual orientation and race, ethnicity and racism.

Reporting on sex and gender	No human participants were invovled.
Reporting on race, ethnicity, or other socially relevant groupings	No human participants were invovled.
Population characteristics	No human participants were invovled.
Recruitment	No human participants were invovled.
Ethics oversight	No human participants were invovled.

Note that full information on the approval of the study protocol must also be provided in the manuscript.

Field-specific reporting

Please select the one below that is the best fit for your research. If you are not sure, read the appropriate sections before making your selection.

☒ Life sciences ☐ Behavioural & social sciences ☐ Ecological, evolutionary & environmental sciences

For a reference copy of the document with all sections, see nature.com/documents/nr-reporting-summary-flat.pdf

Life sciences study design

All studies must disclose on these points even when the disclosure is negative.

Sample size	The N number for all experiments, including animal experiments and in vitro experiments were listed in the figure legends. The sample size was chosen based on our prior studies (PMID: 34615377, 34397273) and other previous papers with similar experiments (PMID: 28924165, 25628421, 36158197), which showed sufficient statistical power for in vitro experiments and animal experiments.
Data exclusions	No samples or animals were excluded from analyses.
Replication	All animal experiments were repeated at least twice and in vitro experiments were repeated at least three times. All results are reproducible and representative data were showed in the figures or supplementary files.
Randomization	Animals were allocated to their respective group at birth by a blinded investigator. For other experiments, including cell experiments, before performing the corresponding treatment, samples were randomly assigned to control and experimental groups by an investigator blinded to subsequent experimental information. The standard laboratory procedures were strictly followed to keeping the experimental environment and facilities consistent and performed under the same conditions.
Blinding	Investigators were blinded to group allocation during data collection, image quantification and data analysis.

Reporting for specific materials, systems and methods

We require information from authors about some types of materials, experimental systems and methods used in many studies. Here, indicate whether each material, system or method listed is relevant to your study. If you are not sure if a list item applies to your research, read the appropriate section before selecting a response.

Materials & experimental systems

n/a	Involved in the study
☐	☒ Antibodies
☐	☒ Eukaryotic cell lines
☒	☐ Palaeontology and archaeology
☐	☒ Animals and other organisms
☒	☐ Clinical data
☒	☐ Dual use research of concern
☒	☐ Plants

Methods

n/a	Involved in the study
☒	☐ ChIP-seq
☐	☒ Flow cytometry
☒	☐ MRI-based neuroimaging

Antibodies

Antibodies used	SLC35D3 (Thermo scientific, #PA572721), Myc (Abcam, #Ab206486; CST, #2276S), His (CST, 12698S), Hes1 (Santa, #sc-166410), NOTCH1 (CST, #3608S; #4380S), NICD (CST, #4147S), Ucp1 (Abcam, #ab10983), Pgc1α (Proteintech, #66369-1-Ig), Prdm16 (Abcam, #ab303534), Na/K ATPase (Abcam, #ab76020; #ab7671), Calnexin (Thermo scientific, # MA3027), Tnf-α (Proteintech, #60291-1-Ig), Ccl2 (Proteintech, #66272-1-Ig), Il6 (Huabio, #EM1701-45), F4/80 (Proteintech, #28463-1-AP), Gapdh (Proteintech, #60004-1-Ig), PE-conjugated Notch1 (CST, #15004S), Alexa Fluor™ 488 Goat anti-Mouse IgG (Invitrogen, #A-11029), Alexa Fluor™ 594 Goat anti-Mouse IgG (Invitrogen, # A-11005), Alexa Fluor™ 488 Goat anti-Rabbit IgG (Invitrogen, #A-11034), Alexa Fluor™ 594 Goat anti-Rabbit IgG (Invitrogen, #A-11012), HRP-conjugated ACTB (Proteintech, #HRP-60008), HRP-conjugated anti-rabbit IgG (Proteintech, # SA00001-2), HRP-conjugated anti-mouse IgG (Proteintech, # SA00001-1), HRP-conjugated anti-rat IgG (Proteintech, # SA00001-15).
Validation	The antibodies including SLC35D3, Myc, His, Hes1, Ucp1, Pgc1α, Prdm16, Na/K ATPase, Calnexin, Tnf-α, Ccl2, Il6, F4/80, Gapdh, HRP-conjugated ACTB, NOTCH1, NICD were validated for the western blotting of both human and mouse samples on the websites of the associated companies (https://www.thermofisher.cn/, https://www.scbt.com/home, https://huabio.com.cn/, https://www.ptgcn.com/, https://www.cellsignal.cn/, https://www.abcam.cn/). Ucp1 and F4/80 antibodies were validated for the immunohistochemical staining of mouse samples on the websites of the companies (https://www.ptgcn.com/,https://www.abcam.cn/). The antibodies including SLC35D3, Myc, NOTCH1, Na/K ATPase and Calnexin were validated for the immunofluorescence staining of both human and mouse samples on the websites of the associated companies (https://www.thermofisher.cn/, https://www.cellsignal.cn/, https://www.abcam.cn/). The PE-conjugated Notch1 was validated for flow cytometry using human samples on the websites of the associated company (https://www.cellsignal.cn/).

Eukaryotic cell lines

Policy information about cell lines and Sex and Gender in Research

Cell line source(s)	HEK293T (ATCC, CRL-3216) were obtained from ATCC.
Authentication	Authentication of the cell line were performed by a Human STR Profiling Cell Authentication Service (ATCC).
Mycoplasma contamination	Cells tested negative for mycoplasma contamination.
Commonly misidentified lines (See ICLAC register)	No misidentified lines were used in the study.

Animals and other research organisms

Policy information about studies involving animals; ARRIVE guidelines recommended for reporting animal research, and Sex and Gender in Research

Laboratory animals	Slc35d3-Flox mice and Slc35d3-WT mice with C57BL/6J background were purchased from Beijing Biocytogen Incorporated Company. Adiponectin-Cre mice and ob/ob mice were purchased from Cyagen Biosciences (Suzhou, China). The mice were housed under a 12-hour light/dark cycle at a temperature of 23 ± 1 °C and relative humidity of 50%-60%, with free access to water. Unless mentioned otherwise, 12-week-old mice were used for experiments.
Wild animals	The study did not involve any wild animal.
Reporting on sex	The experiments in the study were done in both sexes. Relevant information is described detailly in the manuscript.
Field-collected samples	No field-collected samples were used in the study.
Ethics oversight	All animal use and welfare adhered to the National Institutes of Health’s Guide for the Care and Use of Laboratory Animals following a protocol reviewed and approved by the State Key Laboratory of Cardiovascular Disease, National Center for Cardiovascular Diseases, Fuwai Hospital (Beijing, China; permit number: 0000869). The study was reviewed and approved by the ethics committee of Fuwai Hospital (Bejing, China; No. FW-2019-0001).

Note that full information on the approval of the study protocol must also be provided in the manuscript.

Flow Cytometry

Plots

Confirm that:

- [x] The axis labels state the marker and fluorochrome used (e.g. CD4-FITC).
- [x] The axis scales are clearly visible. Include numbers along axes only for bottom left plot of group (a 'group' is an analysis of identical markers).
- [x] All plots are contour plots with outliers or pseudocolor plots.
- [x] A numerical value for number of cells or percentage (with statistics) is provided.

Methodology

Sample preparation	HEK 293T cells were plated in 10 cm plates and transfected with 9 μg of SLC35D3 plasmid per plate using Lipofectamine 3000 transfection reagent. Forty-eight hours post-transfection, the cells were fixed using 4% PFA for 10 min at room temperature, centrifuged at 115 × g for 10 min and washed twice. Next, the cells were incubated for 15 min at 4 °C in FcR blocking medium. Then, the cells were incubated with PE-conjugated Notch1 antibody (CST, 15004) at room temperature for 1 h with rotation, and washed twice. Then 250 μL of ice-cold ligand binding buffer was added to each cell pellet, mixed and passed through the filter cap of a 5 mL polystyrene round-bottom tube. This process removed clumped cells immediately prior to flow cytometry. The MFI of the R-phycoerythrin (PE)-labeled antibody emitting fluorescence at 660 nm was determined with a FACSCalibur (BD Biosciences).
Instrument	FACSCalibur (BD Biosciences)
Software	FlowJo CE 7.5.110.7
Cell population abundance	No cell sorting was performed.
Gating strategy	Single cells were selected by FSC/SSC gates and then FSC/FSC-width, and cells were gated to exclude dead cells, doublets and triplets. Samples were analyzed using the following gating strategy: Notch1 expression on the cell membrane. The gating strategy is also shown in Supplementary Figure 7h.

- [x] Tick this box to confirm that a figure exemplifying the gating strategy is provided in the Supplementary Information.
